# A Phase I trial of the anti-IL23 monoclonal antibody tildrakizumab, in combination with abiraterone acetate, for the treatment of metastatic castration-resistant prostate cancer

**DOI:** 10.1007/s10637-026-01619-x

**Published:** 2026-05-16

**Authors:** Khobe Chandran, Christina Guo, Simon Pacey, Guillermo Villacampa, Ruth Matthews, Toby Prout, Joan Fernandes, Alison Turner, Mona Parmar, Ruth Riisnaes, Ana Ferreira, Suzanne Carreira, Claudia Bertan, Mateus Crespo, Ines Figueiredo, Wei Yuan, Denisa Bogdan, Florence Raynaud, Ruth Ruddle, Bianca Calì, Martino Maddalena, Arianna Calcinotto, Christina Yap, Adam Sharp, Andrea Alimonti, Johann De Bono, Alec Paschalis

**Affiliations:** 1 Institute of Cancer Research, 15Cotswold RoadSM2 5NG, London, UK; 2 The Royal Marsden NHS Foundation Trust, London, UK; 3 Department of Oncology, School of Clinical Medicine, University of Cambridge, Cambridge, UK; 4 Cambridge University Hospitals NHS Foundation Trust, Addenbrookes Hospital, Cambridge, UK; 5 Oncology Institute of Southern Switzerland, Ente Ospedaliero Cantonale, Bellinzona, Switzerland; 6 Institute of Oncology Research (IOR), Bellinzona, Switzerland; 7 Faculty of Biomedical Sciences, Università Della Svizzera Italiana, Lugano, Switzerland; 8 Department of Health Sciences and Technology, Eidgenössische Technische Hochschule Zürich (ETH), Zurich, Switzerland; 9 Department of Medicine, University of Padova, Padua, Italy

**Keywords:** Prostate Cancer, Metastatic Castration-Resistant Prostate Cancer, Treatment Resistance, Interleukin-23, Androgen Receptor Signaling, Phase I trial

## Abstract

**Background:**

Interleukin-23 (IL23) has been reported to drive androgen receptor (AR) and JAK2-STAT3 signaling, promoting treatment resistance and disease progression in advanced prostate cancer (PC). We evaluated the safety, tolerability, and antitumor activity of the anti-IL23 monoclonal antibody tildrakizumab in combination with the AR pathway inhibitor (ARPI) abiraterone acetate (AA) in men with ARPI-resistant metastatic castration-resistant PC (mCRPC).

**Methods:**

mCRPC patients of ECOG performance status (PS) ≤ 2, who had previously progressed on first-line ARPI therapy, were treated with tildrakizumab (100 mg, 300 mg, 600 mg; 4-weekly) in combination with AA (YonsaTM, 500 mg daily). The primary objective was to determine the recommended phase 2 dose. Secondary endpoints were elucidation of pharmacokinetics (PK), pharmacodynamics (PD), and antitumor activity.

**Results:**

No dose limiting toxicities (DLTs) were observed, nor any grade ≥ 3 adverse effects (AEs). The most common treatment-related AEs attributable to tildrakizumab were grade 1—2 fatigue (n = 3/12; 25.0%) and grade 1 nausea (n = 2/12; 16.7%). PK analyses showed no obvious drug-drug interactions. Overall, no objective responses were observed. Median progression free survival (PFS) was 3.7 months (n = 10; 95% CI 1.6-not reached) with median overall survival (OS) of 9.7 months (n = 10; 95% CI 6.9-not reached).

**Conclusions:**

Tildrakizumab and abiraterone acetate combination therapy is well tolerated but did not show clinical efficacy in this small, unselected, cohort of ARPI-resistant mCRPC patients. Further study into the causes of primary resistance to IL23 targeting in mCRPC, and development of biomarkers of downstream IL23-IL23R activity, are now needed to translate IL23 blockade into the clinic.

**Supplementary Information:**

The online version contains supplementary material available at 10.1007/s10637-026-01619-x.

## Introduction

All patients with advanced prostate cancer (PC) eventually develop resistance to currently approved hormonal therapies, termed castration-resistant PC (CRPC), which is invariably fatal. Consequently, PC remains a leading cause of cancer death in men worldwide. Deregulated androgen receptor (AR) signaling is reported to remain a key driver of CRPC and can be induced by genomic alterations of the AR locus, AR splice variants, activation of AR co-regulators, microbiota metabolites, as well as paracrine factors. In addition, non-canonical molecular signaling pathways can also be co-opted by PC cells to reactivate AR signaling and promote cell survival and proliferation, some of which are regulated by factors within the tumor microenvironment (TME).

We and others have previously reported that there is a significant expansion of myeloid-derived suppressor cells (MDSCs) during PC progression, especially to metastatic CRPC (mCRPC), making MDSCs a prominent immune cell subset in the CRPC TME [1]. MDSCs are a heterogeneous population of activated immune cells that can drive nitric oxide synthase (NOS) upregulation and T cell nutrient depletion to create a potent immunosuppressive TME, enabling tumor growth and immune evasion [1]. Furthermore, MDSC-derived factors such as interleukin-23 (IL23) can also fuel PC growth through the STAT3-RORγ axis by promoting angiogenesis, senescence evasion, and stimulating AR and AR-target gene expression [2] [3]. Taken together, preclinical and clinical data support the investigation of treatments targeting IL23-mediated PC progression as a novel therapeutic strategy for reversing resistance to anti-androgen therapies and improving outcomes for patients with lethal mCRPC.

Tildrakizumab is a well-tolerated anti-IL23 monoclonal antibody that can inhibit IL23 activity, with FDA approval for the treatment of psoriasis. Here we present the results of a Phase I trial of tildrakizumab in combination with the AR pathway inhibitor (ARPI) abiraterone acetate (Yonsa™) in men with endocrine treatment-resistant mCRPC.

## Materials and methods

### Patients

Patients aged ≥ 18 years with histologically confirmed mCRPC and Eastern Cooperative Oncology Group (ECOG) performance status 0—2 with disease progression after at least one ARPI were eligible.

### Trial oversight

This investigator-initiated trial was supported by a grant from Sun pharmaceutical, endorsed by Cancer Research UK, and co-sponsored by the Royal Marsden NHS Foundation Trust and the Institute of Cancer Research. It received ethical approval from the NRES Committee London, Surrey Borders.

The Institute of Cancer Research Drug Development Unit Investigator-Initiated Trials (DDU-IIT) team in Sutton, London, had responsibility for all aspects of trial management and statistical analysis. The Trial Management Group oversaw day-to-day trial conduct with strategic oversight provided by an independent trial steering committee. Safety data were reviewed, and dose-escalation decisions made, by the trial Safety Review Committee.

### Study Objectives

The primary aim was to describe the safety and tolerability of tildrakizumab in combination with abiraterone acetate and establish a recommended phase 2 dose (RP2D). Secondary objectives were to evaluate the antitumor activity, pharmacokinetics (PK), and pharmacodynamics (PD) of tildrakizumab and abiraterone acetate in men with mCRPC.

### Study design and treatment

This was a phase I open-label, multicenter, trial of the anti-IL23 monoclonal antibody tildrakizumab in combination with abiraterone acetate (Yonsa™) in patients with mCRPC progressing after at least one ARPI. The study utilized a one-stage Bayesian Continual Reassessment Method (CRM) design [4–6], exploring escalating doses of tildrakizumab (100 mg, 300 mg, 600 mg, IV q4week), in combination with abiraterone acetate (500 mg, oral QD) to identify a RP2D [Supplementary Table 1**]**. To minimize the risk of misinterpreting a rare response to abiraterone re-challenge as re-sensitization mediated by IL23 blockade, all participants underwent a run-in period on abiraterone to confirm resistance by prostate-specific antigen (PSA) progression before commencing combination treatment. However, participants who had received abiraterone within 6-months prior to study entry did not require this run‑in and instead commenced combination treatment upfront.

### Assessments

Safety and tolerability were assessed from the time of first dose to 30 days after treatment discontinuation using adverse event (AE) reporting according to Common Terminology Criteria for Adverse Events (CTCAE) version 4.0. Response assessments used PSA, bone scan, RECIST v1.1, and circulating tumor cell (CTC) counts, and was defined as: PSA decline ≥ 50% from baseline, objective response according to RECIST v1.1, or CTC count conversion from ≥ 5/7.5 ml blood at baseline to < 5/7.5 ml blood.

### Statistical analysis of clinical data

Statistical analysis was descriptive. AEs were tabulated and the proportion of patients with grade 3/4 toxicities and the number and type of serious adverse events (SAEs) were reported. Patients who received at least one dose of tildrakizumab in combination with abiraterone acetate were included in the safety analysis. Patients who received ≥ 8-weeks of combination therapy were included in response analysis. Response rates were calculated with a 95% confidence interval (CI).

### Research sample collection and analysis

Blood samples for PK analyses were obtained as described in ***supplementary methods***, to determine maximum concentration (C_max_), mean residence time (MRT), time to reach maximum concentration (T_max_), terminal elimination half-life (t1/2), drug exposure (AUC) and volume of distribution (V). Where feasible, paired mCRPC tissue biopsies were collected prior to and 4-weeks after commencement of combination therapy for PD analyses. STAT3 and pSTAT3 protein levels were evaluated by immunohistochemistry (IHC) as described in ***supplementary methods*** and quantitated by a pathologist blinded to clinical data. RNA sequencing (RNAseq) was performed as per previously reported methods [7]. IL6_JAK_STAT3_SIGNALLING and INFLAMMATORY_RESPONSE RNA activity signatures were obtained from the molecular signatures database *(Hallmark)* [8]. AR activity score was generated using previously published methods [7].

## Results

### Patients

Overall, 13 patients received at least one study drug from February 2022 to August 2022 **[**Table 1**]*.*** All participants had previously received one systemic therapy for mCRPC at the time of enrollment, with most having received two or more prior lines of treatment (n = 9; 69.2%), including abiraterone (n = 6, 46.2%), enzalutamide (n = 5, 38.5%), docetaxel (n = 7, 53.8%) and cabazitaxel (n = 5, 38.5%). One patient developed disease progression during the abiraterone run-in phase and did not receive tildrakizumab; this patient was therefore excluded from the safety analysis. The remaining 12 participants comprised the safety population. Of these, one did not receive at least 80% of the planned doses of abiraterone in cycle one, and one discontinued treatment due to clinical progression having only received one dose of tildrakizumab. Consequently, 10 participants were eligible for response evaluation **[**Supplementary Fig. 2**]**.

**Table 1:** Participant baseline clinical characteristics

			**Overall (n = 13)**
Age (years; mean and range)			74.2 (67.0–83.3)
Histology		Adenocarcinoma	13 (100%)
Primary tumour stage at diagnosis, n (%)		T2	1 (9.1%)
		T3	6 (54.5%)
		T4	4 (36.4%)
		TX	2 (16.7%)
Lymphadenopathy at diagnosis, n (%)		N0	4 (30.7%)
		N1	8 (61.6%)
		NX	1 (7.7%)
Metastatic disease at diagnosis, n (%)		Yes	6 (46.2%)
		No	7 (53.8%)
Gleason score at diagnosis		5	1 (7.7%)
		6	1 (7.7%)
		7	2 (15.4%)
		8	1 (7.7%)
		9	7 (53.8%)
		Not available	1 (7.7%)
Disease stage		3B	1 (7.7%)
		4	12 (92.3%)
WHO performance status		PS 0	3 (23.1%)
		PS 1	10 (76.9%)
Prior treatment	**At diagnosis**	Prostatectomy	3 (23.1%)
		Radiotherapy	2 (15.4%)
		Systemic treatment	8 (61.5%)
	**mCRPC**	Chemotherapy	8 (61.5%)
		ARPI	13 (100.0%)
		Other	5 (38.5%)
	**Number of lines at mCRPC**	1	4 (30.8%)
		2	3 (23.1%)
		3	2 (15.4%)
		4	1 (7.7%)
		≥ 5	3 (23.1%)

### Safety and tolerability

Combination therapy with tildrakizumab and abiraterone was well-tolerated with no new safety signal. 157 AEs were reported in total, with 16.6% (26/157) of those judged to be treatment related. Seven patients experienced at least one treatment-related AE **[**Table 2**].** The most common AEs related to tildrakizumab were fatigue (25.0%) and nausea (16.7%). In keeping with previous reports [7], the most common AEs related to abiraterone were fatigue (30.8%), hypokalemia (15.4%), and nausea (15.4%). Overall, all AEs were grade 1—2 in severity with no reported SAEs or dose-limiting toxicities (DLTs)**.**

**Table 2:** Treatment-emergent adverse events related to tildrakizumab and abiraterone

	All grades		Grade 1 or 2		Grade 3 or 4		
Adverse event to tildrakizumab	n	%	n	%	n		%
Fatigue	3	25%	3	25	0		0
Nausea	2	16.7%	2	16.7	0		0
Vomiting	1	8.3%	1	8.3	0		0
Neutropenia	1	8.3%	1	8.3	0		0
Anemia	1	8.3%	1	8.3	0		0
Aspartate aminotransferase increased	1	8.3%	1	8.3	0		0
Decreased appetite	1	8.3%	1	8.3	0		0
	**All grades**		**Grade 1 or 2**		**Grade 3 or 4**		
Adverse event related to abiraterone	n	%	n	%	n	%	
Fatigue	4	30.8	4	30.8	0	0	
Hypokalemia	2	15.4	2	15.4	0	0	
Nausea	2	15.4	2	15.4	0	0	
Decreased appetite	1	7.7	1	7.7	0	0	
Vomiting	1	7.7	1	7.7	0	0	
Neutropenia	1	7.7	1	7.7	0	0	
Aspartate aminotransferase increased	1	7.7	1	7.7	0	0	
Atrial fibrillation	1	7.7	1	7.7	0	0	

### Antitumor activity

**Fig. 1:**
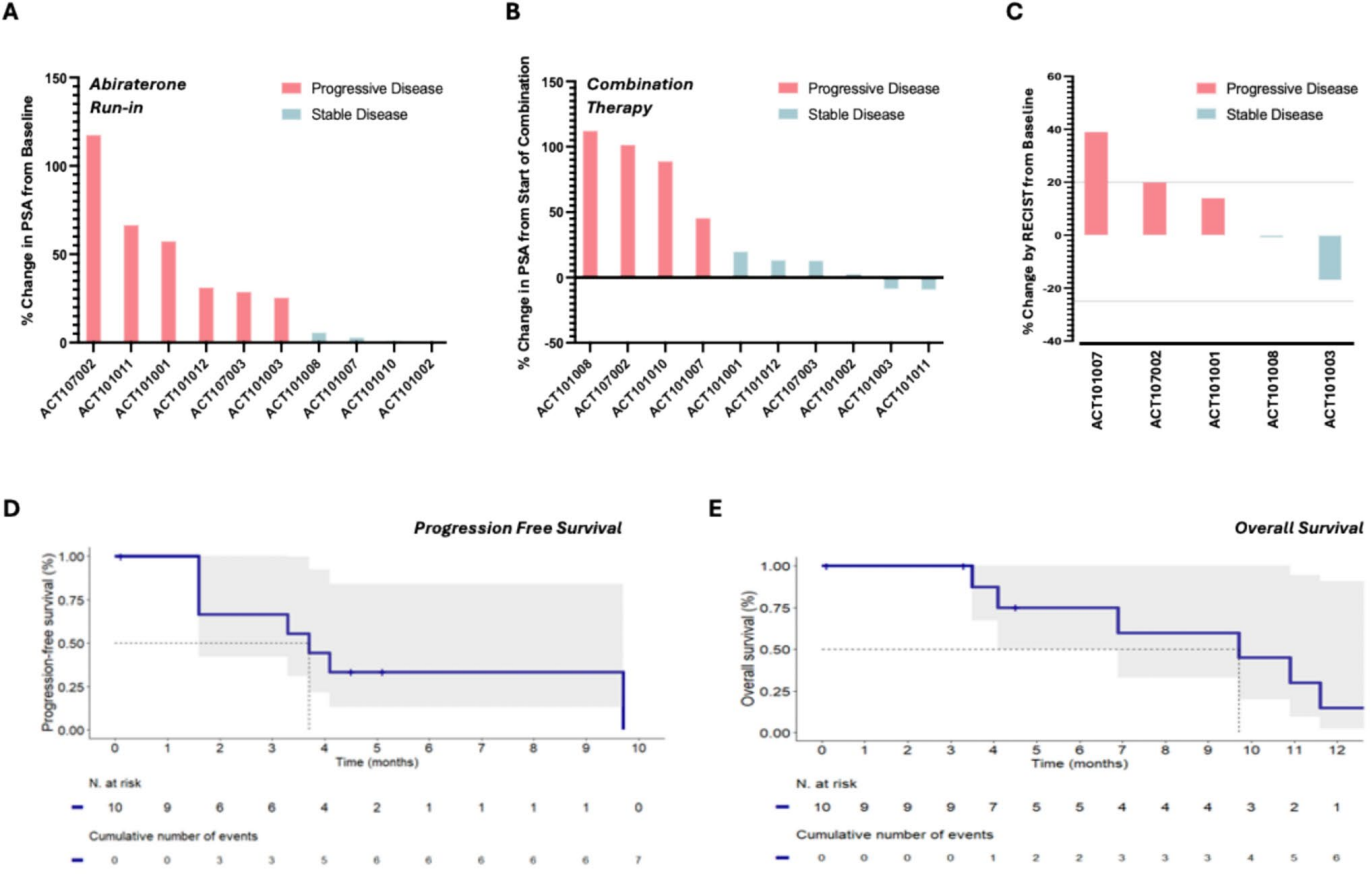
Antitumor activity assessments of response evaluable participants with metastatic CRPC treated with combination tildrakizumab and abiraterone acetate. (**A**-**B**) Waterfall plots of PSA response in the abiraterone run-in period (**A**) and best PSA response following addition of tildrakizumab (**B**). (**C**) Waterfall plot of best radiological response per RECIST v1.1 in evaluable participants (n = 5). (**D-E**) Kaplan–Meier curves demonstrating progression-free (**D**) and overall (**E**) survival for all evaluable patients (n = 10)

10 patients completed at least 8-weeks of study treatment and were eligible for response evaluation. All 10 of these patients were evaluable by PSA response **[**Fig. 1A-B**]** and CTC enumeration **[**Supplementary Fig. 3**]**, with 5 evaluable by RECIST v1.1 **[**Fig. 1C**]**. Among the 10 patients evaluable by CTCs, the median CTC count at baseline was 44.5 (IQR 12.5—94.5). No responses were identified by CTC values. Overall, no patients demonstrated an objective complete or partial response to treatment. Median progression free survival (PFS) was 3.7 months (n = 10; 95% CI 1.6-not reached) with a median overall survival (OS) of 9.7 months (n = 10; 95% CI 6.9-not reached) **[**Fig. 1C-D**].**

### Pharmacokinetic Profile

**Fig. 2:**
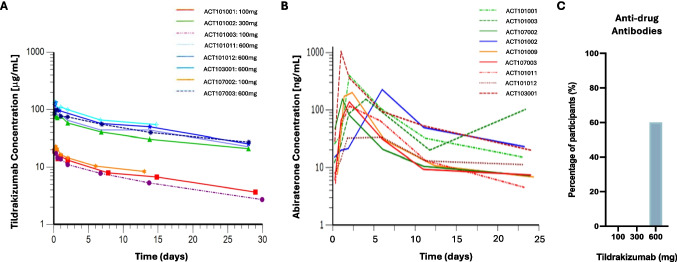
Pharmacokinetic evaluation of tildrakizumab and abiraterone acetate. (**A**) Line graph demonstrating the change in tildrakizumab concentration over time. Blood samples collected between day 1 and day 29 of combination treatment (pre-second dose) in patients treated at doses of 100 mg (n = 3), 300 mg (n = 1), and 600 mg (n = 4). (**B**) Line graph demonstrating the pharmacokinetic profile of abiraterone acetate dosed daily at 500 mg PO during the single agent abiraterone confirmation of resistance run-in (n = 9). (**C**) Bar graph indicating percentage of participants with detectable anti-drug antibodies at each dose level (n = 9)

Nine patients had sufficient PK sampling performed to enable PK profiling. Evaluation of plasma tildrakizumab levels demonstrated a dose-dependent increase in total drug exposure (AUC_0-∞_), with an AUC_0–24_ and C_max_ comparable to previous studies where healthy volunteers have been given a single 200 mg IV dose **[**Fig. 2A**].** PK sampling for abiraterone acetate, collected at the start of the run-in period and pre-dose on C1D1 and C2D1 of the combination treatment, revealed drug levels comparable to historical data for the same formulation **[**Fig. 2B**],** with no significant change in drug levels (C_max_ and AUC_last_) before and after the commencement of tildrakizumab (data not shown). Taken together, these data do not support the presence of any drug-drug interaction between abiraterone and tildrakizumab. The presence of antibody–drug antibodies (ADAs) was also evaluated across dose levels. ADAs were not detected in any patients receiving doses of 100 mg or 300 mg tildrakizumab. However, three out of five patients (60%) receiving the 600 mg dose had detectable levels of ADAs. These emerged as early as C1D8 but were not consistently detectable at subsequent time-points **[**Fig. 2C**]**.

### Pharmacodynamic profile

**Fig. 3:**
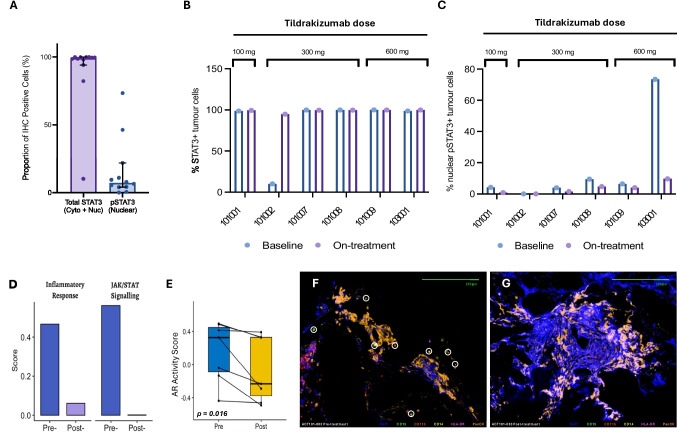
Pharmacodynamic evaluation of combination tildrakizumab and abiraterone acetate therapy. (**A**) Proportion (%) of tumor cells in participant metastatic CRPC (mCRPC) tissue biopsies staining positive by immunohistochemistry (IHC) for STAT3 and pSTAT3 at baseline, per participant treated with tildrakizumab and abiraterone (**B**) Proportion (%) of tumor cells staining positive by IHC for STAT3 before and after treatment with tildrakizumab plus abiraterone acetate (500 mg daily) in participants with sufficient mCRPC tissue for evaluation. (**C**) Proportion (%) of tumor cells staining positive by IHC for pSTAT3 before and after treatment with tildrakizumab plus abiraterone acetate (500 mg daily) in subject with sufficient mCRPC tissue for evaluation. (**D**) RNA-sequencing analysis of IL6_JAK_STAT3 (JAK/STAT; *Hallmark*) and INFLAMMATORY_RESPONSE *(Hallmark)* pathway signaling in matched, same-patient, mCRPC tissue biopsies taken prior to and after treatment with tildrakizumab and abiraterone in subject 103001, who exhibited a reduction in pSTAT3 with treatment (n = 1). **(E)** RNA-sequencing analysis of AR activity score [7] in matched, same-patient, mCRPC tissue biopsies taken prior to and after treatment with tildrakizumab and abiraterone (n = 7). (**F**-**G**) Representative micrographs from subject 101003 prior to (F) and following (G) treatment with tildrakizumab and abiraterone, demonstrating a reduction in PMN-MDSCs (white circles) following treatment. Scale bars = 200 μm. *Abbreviations: Pre – prior to treatment; Post – following treatment*

In the absence of a single validated, direct biomarker of IL23 signaling pathway activity, exploratory analyses evaluating the PD effects of tildrakizumab were assessed by measuring changes in STAT3 and pSTAT3 protein levels in available mCRPC tissue samples collected before and after treatment with abiraterone acetate and tildrakizumab (n = 6), given that IL23-mediated signal transduction through the IL23R is reported to activate JAK/STAT signaling [9]. At baseline, STAT3 protein expression was present across most tumor samples (median percentage of cells positive = 99% [Interquartile range [IQR] 95—100%]), while pSTAT3 expression was scattered and localized to focal clusters of nuclear pSTAT3 staining (7% [4—19%]) **[**Fig. 3A**]**. Following treatment with tildrakizumab, one patient demonstrated an increase in the proportion of total STAT3 expressing tumor cells, although this patient had fewer total STAT3^+^ tumor cells at baseline. Otherwise, no significant change in STAT3 levels was observed. With regards to pSTAT3 levels, there was a decrease in the proportion of nuclear pSTAT3^+^ tumor cell following treatment, but since few cells were pSTAT3^+^ at baseline this absolute difference was small, except for one patient in whom the proportion of pSTAT3^+^ tumor cells decreased from 73% to 10% post-treatment with tildrakizumab (600 mg) plus abiraterone **[**Fig. 3B-C**]**. In keeping with these findings, RNAseq analysis demonstrated upregulated baseline IL6_JAK_STAT3 and INFLAMMATORY_RESPONSE signatures *(Hallmark)* in this participant’s baseline mCRPC tissue, with downregulation of these pathways following combination therapy **[**Fig. 3D**]**. Notably, in this patient, after an initial PSA rise from 337.2 to 445.0 on C2D1 of combination therapy, PSA subsequently declined to 325.9 on C2D8. However, the patient discontinued treatment due to clinical disease progression after receiving only a single dose of tildrakizumab and was therefore not response evaluable.

Consistent with this observed reduction in PSA, analysis of available RNAseq data (n = 7) revealed an overall reduction in AR activity score following tildrakizumab treatment **[**Fig. 3E**]**, although no other significant signaling pathway changes were identified.

By contrast, evaluation of mCRPC tissue samples collected from participant 101003, who exhibited the greatest RECIST response, revealed a marked reduction in intra-tumoral PMN-MDSC infiltration following combination therapy compared with baseline **[**Fig. 3F-G**]**.

Taken together, however, these data do not provide conclusive evidence that this therapeutic strategy consistently modulates IL23 signaling.

## Discussion

This study arguably represents the first investigation of IL23 inhibition in cancer, with tildrakizumab administered up to three times previously administered doses for treating psoriasis. Importantly, no DLT or new safety signal was observed, with no identified PK interactions. However, it remains unclear whether tildrakizumab effectively blocks IL23R-JAK2-STAT3 signaling in PC. Overall, there was no objective response in the 10 unselected response-evaluable ARPI-resistant mCRPC patients.

Several factors may account for this lack of antitumor activity, including the choice and sequencing of the combination partner, as well as the advanced, heavily pretreated nature of the study population. Although preclinical studies suggest that IL23 antagonism can increase the efficacy of enzalutamide [2], both advanced disease and AR pathway inhibition exert complex, context-dependent immunologic effects, including alterations in intratumoral myeloid cell populations and T-cell function, which may contribute to a more immunosuppressive tumor immune microenvironment and limit the therapeutic impact of IL23 blockade. The observed lack of antitumor activity therefore prompts inquiry into the causes of primary resistance to IL23 targeting in mCRPC. Whilst preclinical studies involving xenograft and transgenic mouse models have suggested that single-cytokine targeting or deletion can prevent carcinogenesis and control tumor growth [2, 10–13], monotherapies antagonizing cytokines (e.g. TNF, IL-6/IL-6R, IL-1β/IL-1R, TGF-β/TGF-βRI) have generally yielded limited clinical success in oncology [14]; although there is emerging evidence of their synergy when combined with other immunotherapies such as T cell engagers or immune checkpoint inhibitors [9]. This stands in contrast to other inflammatory pathologies, such as psoriasis, autoimmune arthropathies, and inflammatory bowel disease, where single cytokine targeting has shown clinical efficacy [9]. This may in part be because the degree of pathway inhibition needed to impact tumor growth remains uncertain, given that in vivo experiments typically utilize IL23 genetic knockout models [2]. For this reason, we pursued a significantly higher dose of tildrakizumab than that used for inflammatory disorders where normalization rather than blockade of dysfunctional signaling was likely adequate. The ability of tildrakizumab to penetrate prostate tumors is also unclear; this is particularly relevant because the source of IL23 in CRPC is paracrine, making inhibition of circulating IL23 inadequate [2]. Future early phase studies could incorporate anti-drug IHC to confirm intratumor biodistribution. Beyond these practical considerations, the comparison with efficacy in non-malignant diseases highlights the complex pathophysiology of cancer associated inflammation, where redundant networks of multiple cytokines are involved. For example, IL6, IL23, and, to some extent, IL12, all engage the JAK2-STAT3 pathway [15]. The lack of an IL23-IL23R specific biomarker readout is therefore a major limitation in the PD evaluation of tildrakizumab in this study, as it was not possible to determine by IHC whether the lack of substantial pSTAT3 downregulation was due to lack of baseline target expression, inadequate IL23R inhibition, or redundant IL12 and IL6 signaling. Consequently, inferences regarding IL23 pathway modulation based on changes in pSTAT3 alone should be interpreted with caution.

Another key challenge to cytokine targeting in cancer is that it remains unclear whether the pathway targeted is a cause or effect of malignancy and its treatment. Cytokine pleiotropy, defined as the ability to exhibit diverse functionalities – including both pro- and anti-tumor effects – may also contribute to resistance. Whilst IL23 has been shown to directly increase PC oncogenic signaling, under physiologic conditions IL23 is produced by activated dendritic cells and macrophages to trigger T helper 17 (TH17) differentiation [16]. TH17 cells have been reported to have paradoxical functions in cancer; the production of IL17A has been shown to promote angiogenesis and immunosuppression. However, they are also important for the recruitment and priming of CD8 + T cells and show plasticity in their ability to differentiate towards IFN-γ producing TH1 cells [17]. Improved understanding of the pathophysiological role of targeted cytokines at an individual patient level is therefore key to realizing the therapeutic utility of targeting cytokine signaling pathways. The analysis presented herein shows marked inter-patient heterogeneity in baseline pSTAT3 expression, with nuclear pSTAT3 only expressed by small clusters of cells in all but one patient, where almost all cells expressed nuclear pSTAT3. Interestingly, the STAT3 signaling pathway was also upregulated at the transcriptional level in this patient. Moreover, this was also the only patient where significant pathway downregulation was observed with treatment. Based on in vitro data that only the neuroendocrine, AR negative, cells constitutively express pSTAT3 [18, 19], the pSTAT3 expressing cell clusters observed may be clusters of cells undergoing differentiation to an AR negative, basal-like phenotype, or responding to paracrine foci. Further studies, for example, using single-cell spatial sequencing are therefore needed to elucidate drivers of focal pSTAT3 activation and its downstream effects. Such studies would also help to determine whether clinically impactful IL23 blockade requires biomarker selection for patients where this pathway is active, or whether combination treatments that stimulate STAT3 pathway activation, such as radiation, are required.

## Conclusion

Co-targeting of IL23 and AR with tildrakizumab and abiraterone acetate is safe, however did not show clinical efficacy in this small, unselected, cohort of ARPI-resistant mCRPC patients. Future clinical trials of IL23‑directed therapies may therefore require biomarker‑driven patient selection to identify patients in whom this pathway is active, in order to adequately evaluate therapeutic potential in mCRPC. Further study into the causes of primary resistance to IL23 targeting in CRPC, and development of biomarkers of downstream IL23-IL23R activity, are now required to facilitate translation of the encouraging preclinical effects of IL23 blockade into the clinic.

## Supplementary Information

Below is the link to the electronic supplementary material.

Supplementary file1 (PDF 1.69 MB)

## Data Availability

The datasets used in the current study may be made available from the corresponding author upon reasonable request, subject to approval from the trial Principle Investigator (PI) and Sponsor.
