# Supplementary material for: A Phase I trial of the anti-IL23 monoclonal antibody tildrakizumab, in combination with abiraterone acetate, for the treatment of metastatic castration-resistant prostate cancer
Source: Invest New Drugs. 2026 May 16;44(4):285–94. doi: 10.1007/s10637-026-01619-x (PMC13486053; doi:10.1007/s10637-026-01619-x)
Supplement: Supplementary file 1 [file 10637_2026_1619_MOESM1_ESM.pdf]

SUPPLEMENTARY DATA

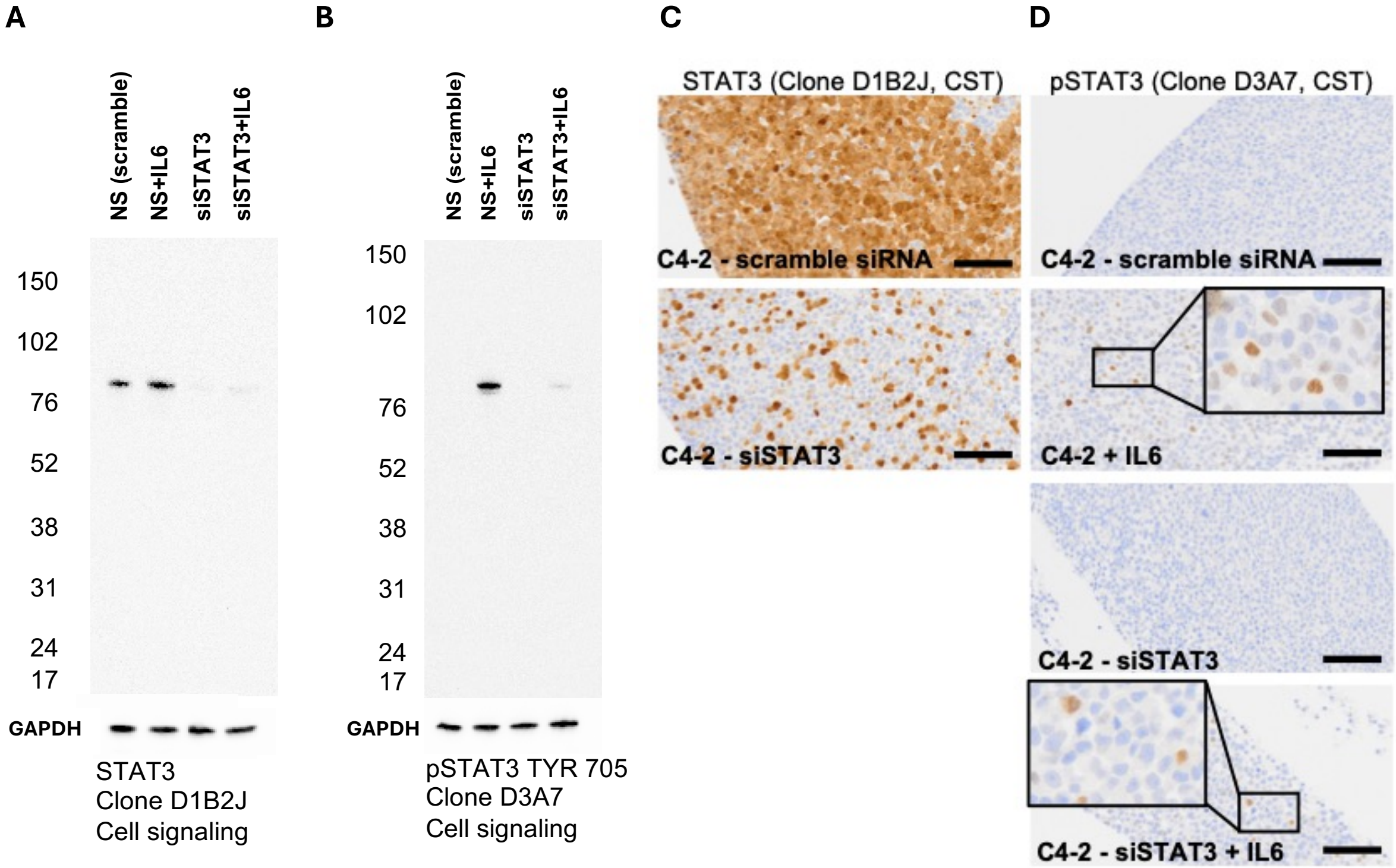

Supplementary Figure 1: Validation of STAT3 and pSTAT3 antibodies used for immunohistochemical (IHC) analyses of metastatic CRPC tissue samples. (A-B) Western blots showing C4-2 prostate cancer cell lysates treated with control (scrambled) siRNA (NS), recombinant human interleukin-6 (IL6) (10 ng/ml), STAT3 siRNA, and STAT3 siRNA plus IL6. Recombinant human IL6 was added 48 hours after siRNA transfection. Protein lysates and cell pellets were collected 72 hours after transfection. (A) shows staining for STAT3 and (B) shows staining for pSTAT3. (C-D) Representative micrographs showing IHC evaluation of STAT3 (C) and pSTAT3 (D) in C4-2 prostate cancer cell pellets following treatment with either control siRNA or STAT3 siRNA +/- IL6. Brown (positive) and blue (negative) staining for STAT3 or pSTAT3 respectively. Scale bars = 100 μm.

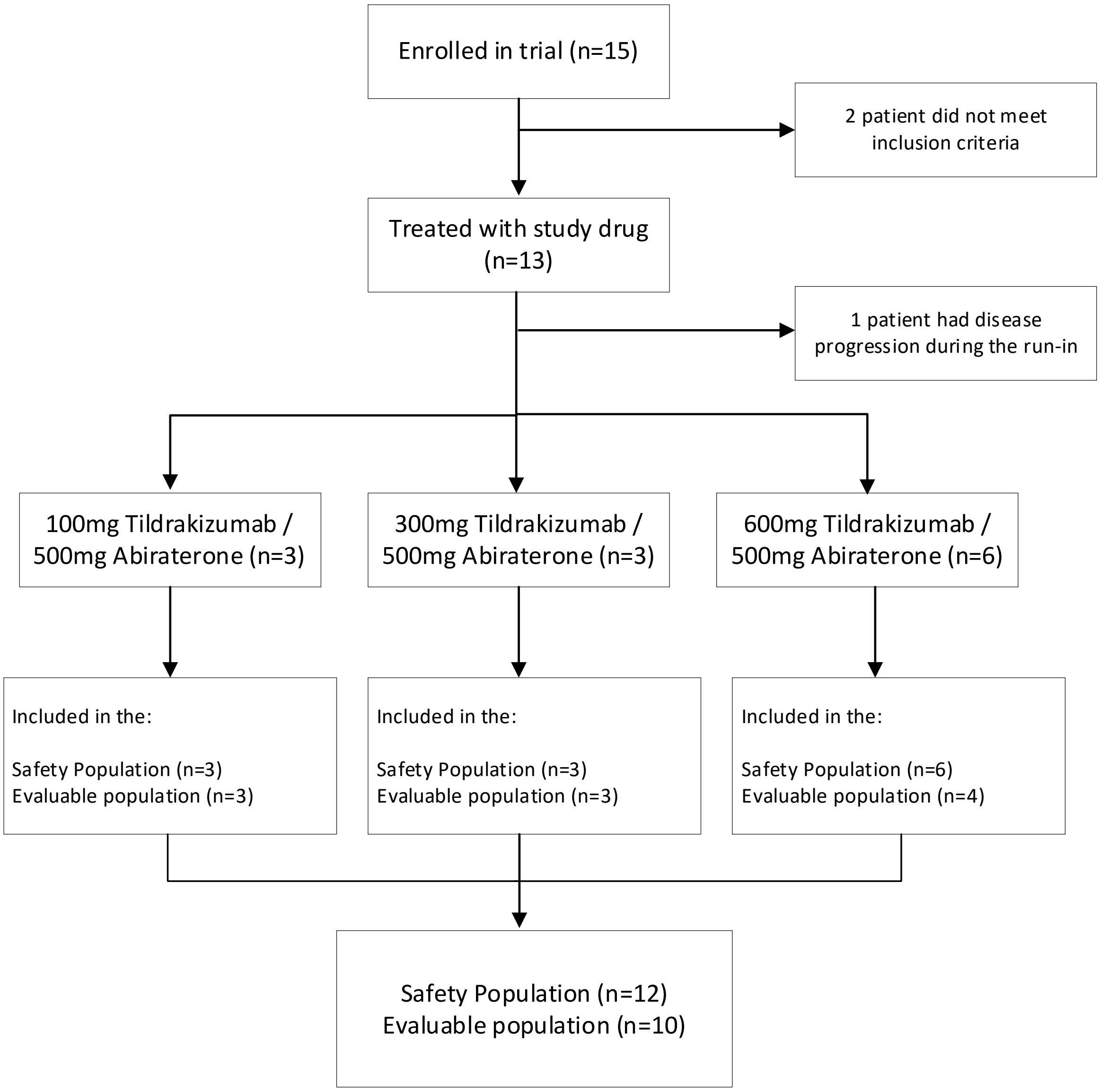

Supplementary Figure 2: Consort diagram for the phase I trial of tildrakizumab in combination with abiraterone acetate for the treatment of metastatic castration-resistant prostate cancer.

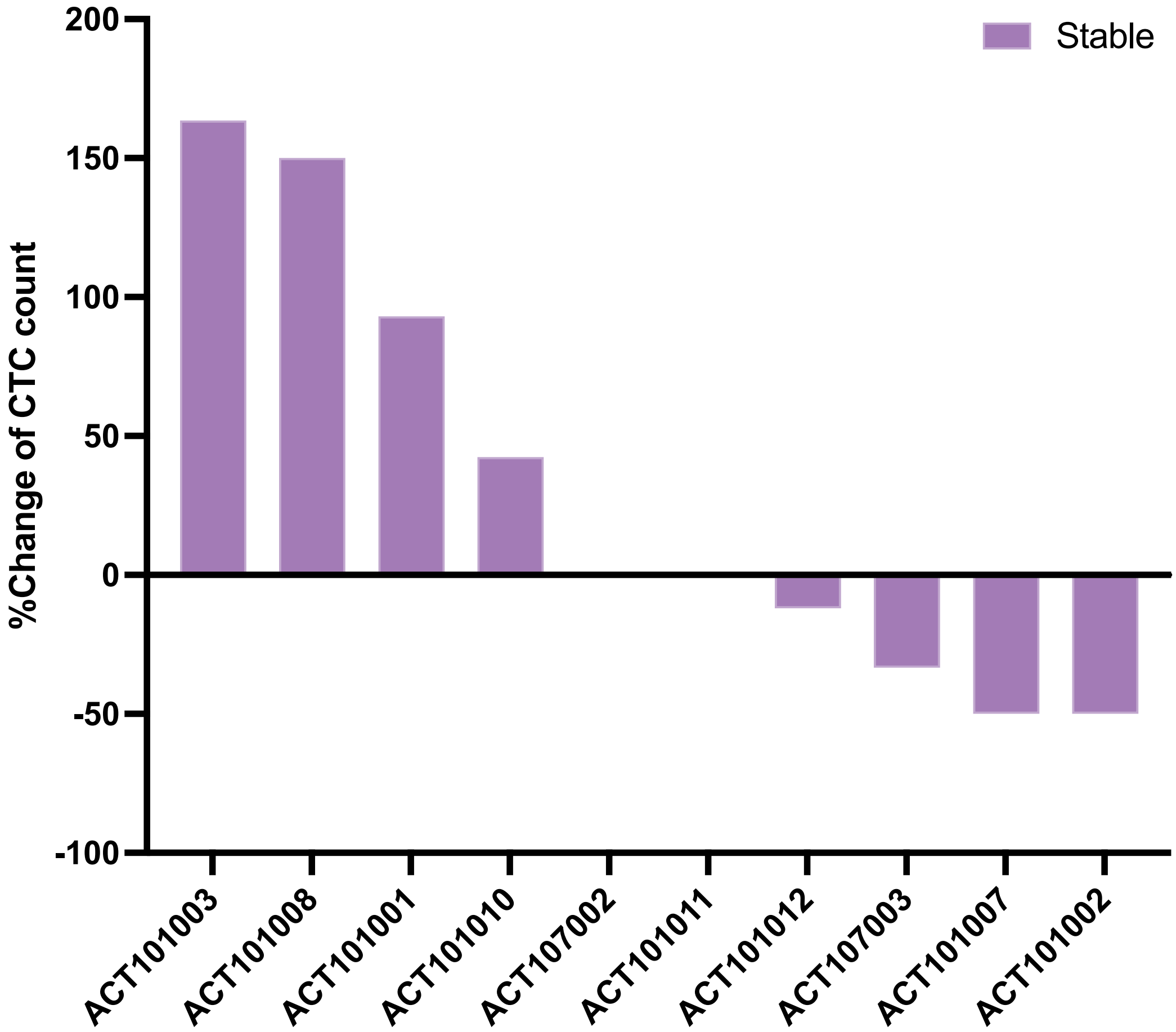

Supplementary Figure 3: Waterfall plot of percentage (%) change from baseline in CTC count following initiation of combination tildrakizumab with abiraterone acetate.

Supplementary Table 1: Dose Escalation Regime

Dose Level	Tildrakizumab (IV every 4 weeks)	Abiraterone<br><br>(Oral daily with prednisolone 5mg bid)	Number of dose limiting toxicities (DLTs)
1	100 mg	500 mg	0
2	300 mg	500 mg	0
3	600 mg	500 mg	0

Supplementary Methods

Research sample collection and analysis

Samples for abiraterone acetate PK analysis were taken during the resistance run-in on cycle 1 day 8 (C1D8) (pre-dose and then 15-minutes, 1-hour, 2-hours, 6-hours, 12-hours post-dose), C1D9 (24-hours post day 8 dose), and on C1D1 and C2D1 of combination treatment with tildrakizumab (pre-dose only). Samples for tildrakizumab PK analysis (combination) were collected on C1D1 (pre-dose and then 2-hours, 6-hours, 12-hours, 24-hours and 48-hours post-dose) and prior to dosing on C1D8, C1D15, C2D1, C2D15, C3D1, C4D1, and C6D1. PK parameters were analysed to determine maximum concentration (Cmax), mean residence time (MRT), time to reach maximum concentration (Tmax), terminal elimination half-life ($t_{1/2}$), drug exposure (AUC) and volume of distribution (V). PK blood samples for tildrakizumab were analysed at Labcorp. PK blood samples for abiraterone acetate were analysed at Sun Pharmaceuticals. PK modelling was conducted using a non-compartmental extravascular model for plasma (200-202) with Phoenix^{TM} WinNonLin Software® version 64 (PhARPIght).

PD analyses were performed on mCRPC tissue samples from patient biopsies collected prior to and, where feasible, 4 weeks after starting combination therapy. STAT3 and pSTAT3 protein levels were evaluated by immunohistochemistry (IHC) using the rabbit anti-STAT3 mAb (Cell Signaling; #30835) and the rabbit anti-phospho-STAT3 mAb (Cell Signaling; #9145). Briefly, antigen retrieval was performed for 10 minutes with Bond ER2 solution (Leica Biosystems), anti-STAT3 antibody (1:250 dilution) and anti-phospho-STAT3 antibody (1:50 dilution) were incubated with tissue for 15 minutes and 30 minutes, respectively. The reaction was visualized using the Bond Polymer Refine (Leica Biosystems) system. Cell pellets from C4-2 cells treated with STAT3 siRNA with and without IL6 were used to confirm specificity of the antibodies by Western blotting and IHC **[Supplementary Figure 1]**. Rabbit IgGs were used as negative controls. The proportion of tumour cells staining for STAT3 (cytoplasmic or nuclear) and pSTAT3 (nuclear), respectively, were algorithmically quantitated by a pathologist who was blinded to clinical data. RNA sequencing (RNAseq) was performed as per previously reported methods [7]. JAK-STAT and inflammatory response RNA activity signatures were obtained from the molecular signatures database *(Hallmark)* [8]. AR activity signatures were generated using previously published methods [7].
